# Single cell genomics of bacterial pathogens: outlook for infectious disease research

**DOI:** 10.1186/s13073-014-0108-0

**Published:** 2014-11-20

**Authors:** Jeffrey S McLean, Roger S Lasken

**Affiliations:** School of Dentistry, Department of Periodontics, University of Washington, Seattle, WA 98195 USA; J. Craig Venter Institute, Capricorn Lane, La Jolla, CA 92037 USA

## Abstract

Genomic sequencing from single cells is a powerful tool in microbiology and holds great promise for infectious disease research. Vast numbers of uncultivable species and pathogens that persist at low abundance in environmental reservoirs are now accessible for genomic analysis.

## Recent advances in single-cell bacterial genomics

DNA sequencing from single cells has revolutionized microbial genomics [1]. The capture of bacterial genomes has been a long-standing challenge in microbiology research because the great majority of bacterial species cannot be readily cultivated. Now, the genomic content of an organism can be sequenced directly from a single cell [2]. The advent of single-cell sequencing sparked a vigorous effort by microbiologists to assemble reference genomes for diverse, uncultivable bacterial species [3]. Until recently, more than half of the 61 currently known phyla in the domain Bacteria were identified only from their 16S rRNA gene sequence. In the past several years, the first reference genomes have been assembled for 18 of these phyla using DNA from single cells, and the remaining candidate phyla are likely to be filled in within the next few years [3].

In 2013, single-cell sequencing was named ‘method of the year’ in recognition of its recent impact on several scientific fields [4]. Single-cell sequencing now holds great promise for research into infectious diseases, where these technologies are just beginning to be employed [3]. It typically involves isolating cells and subsequent amplification of the single copy of the genome by multiple displacement amplification (MDA) [5,6], which makes up to billions of copies of the DNA thereby allowing whole-genome sequencing [2]. In the past, MDA has been used to sequence small quantities of DNA obtained directly from human clinical specimens, such as infected tissue [7], synovial fluid [8] or genital swabs [9]. Although these studies sequenced DNA from multiple cells, they demonstrated that it is technically feasible to analyze the genomes of pathogens taken directly from clinical specimens without the need to culture them, and thus opened the path for single-cell sequencing of pathogens. Several applications of single-cell genomics to the infectious disease field are developing, such as tracking pathogen persistence and transmission, targeted and untargeted pathogen-genome recovery, and the identification of novel bacteria that have pathogenic potential from the human microbiome.

## Applications for the analysis of pathogen persistence and transmission

One potential application of bacterial single-cell genomics is the detection of hospital pathogens during those phases of their life cycle when they persist at very low levels in environmental reservoirs and can be transmitted but not detected easily. Disease-causing organisms, such as *Legionella pneumophila* and *Vibrio cholerae*, are known to reside inside amoeba and biofilms (sometimes within water distribution systems) at barely detectable levels.

The first single-cell pathogen study was of a biofilm isolated from a hospital restroom sink [10,11]. In this application of single-cell genomics, roughly 400 amplified genomes of interest from 25 different genera from the indoor environment of a health care facility were captured using an automated process. Genomic DNA from cells sorted by flow cytometry was amplified using MDA and then screened by 16S rRNA gene polymerase chain reaction to identify taxa of interest for deep sequencing [10,11]. Three individual amplified genomes were obtained for *Porphyromonas gingivalis*, a human pathogen whose genome had previously only been sequenced from cultured isolates from patients. These were the first genomes for this infectious agent to be obtained from a source outside of a human host, with the largest *de novo* assembly being a complete genome [10]. The three independent single *P. gingivalis* cell MDAs were confirmed to be highly clonal with variations in several key virulence factors compared to a host-derived reference.

## Targeted and untargeted pathogen genome recovery

Single-cell genomics can be used both to target specific pathogens and for unbiased screening for population studies and discovery of novel species. A novel promising approach for untargeted genome recovery of a wide array of pathogen genomes is ‘mini-metagenomics’ [11]. This method is intermediate between the use of single-cells and the sequencing of genomes from the thousands of species that can contribute to a metagenomic sample. After cell sorting by flow cytometry, small pools of cells isolated from the environment are amplified by MDA. The reduced diversity of the pools, compared to whole-community metagenomics, makes it simpler to identify and separate individual genomes. This approach was used to randomly screen 18,000 single cells in 288 amplified pools for species of interest. The first genome assemblies were obtained from a member of the uncultivated candidate phylum TM6 [11], demonstrating the ability of the mini-metagenome method to identify rare genomes for sequencing.

## Human microbiome: potential novel pathogen genomes

The development of sampling and single-cell sorting methods for human skin, stool and oral swab samples, combined with the capacity of a high-throughput single-cell genomics platform [10,11], has created new opportunities to capture the genomic diversity of complex microbial communities. The first such study was conducted by the Human Microbiome Project (HMP), which was funded by the National Institute of Health. This project has enabled the submission of genomic sequences from over 400 microbiome bacterial species, many of which were on a list of commensal and potentially pathogenic members of the human microbiome that had no reference genome, referred to as the ‘100 most wanted’ (http://hmpdacc.org/most_wanted/). The HMP initially resulted in genomes for more than 40 species, which were publicly available to the research community. About 145 additional priority genomes are currently being deposited as part of the HMP reference genome set (www.ncbi.nlm.nih.gov/bioproject/28331). Obtaining a complete inventory of genes within human-associated bacterial strains is a crucial step as we seek to understand the role of each of our microbial partners in maintaining health or contributing to disease.

## Looking forward

Recent studies have demonstrated the utility of single-cell genomics for capturing and recovering genomic data from pathogens, and demonstrate progress towards the eventual adoption of this technique in standard clinical applications. By using single-cell genomic strategies, pathogens can be analyzed without prior cultivation, providing a direct unbiased sampling. Current practices of identification after cultivation are restricted to what will grow on the media plate and within a certain time frame. Even when culturing is possible, growth biases can result in selection for genome alterations such as gene loss. Single-cell sequencing of the source organism is desirable to capture all genomic content, including extrachromosomal elements such as plasmids. MDA does not typically provide 100% of the genome from one bacterium [1]. Breaks in the single genome copy lysed from the cell as well as amplification bias from MDA make it necessary to combine data from several different single cells to close a genome assembly completely. However, advances made on the technical and computational fronts have improved genome recovery [3]. There are many examples in which it is necessary to uncover the genomic content of a pathogen that resides at a low level within a host, or that persists in a biofilm or other environmental reservoir. Single-cell sequencing technology has advanced to a stage where this type of research is now highly feasible. We look forward to continued improvements in the laboratory and analytical methods used to date, as well as to exciting new applications in the study of infectious disease and the maintenance of a healthy microbiome.

## Abbreviations

HMP: Human Microbiome Project
MDA: Multiple displacement amplification

## References

[1] Lasken RS (2012). Genomic sequencing of uncultured microorganisms from single cells. Nature Rev Microbiol.

[2] Raghunathan A, Ferguson HR, Bornarth CJ, Song W, Driscoll M, Lasken RS (2005). Genomic DNA amplification from a single bacterium. Appl Environ Microbiol.

[3] Lasken RS, McLean JS (2014). Recent advances in genomic DNA sequencing of microbial species from single cells. Nature Rev Genet.

[4] Chi KR (2014). Singled out for sequencing. Nat Methods.

[5] Dean FB, Nelson JR, Giesler TL, Lasken RS (2001). Rapid amplification of plasmid and phage DNA using Phi 29 DNA polymerase and multiply-primed rolling circle amplification. Genome Res.

[6] Dean FB, Hosono S, Fang L, Wu X, Faruqi AF, Bray-Ward P, Sun Z, Zong Q, Du Y, Du J, Driscoll M, Song W, Kingsmore SF, Egholm M, Lasken RS (2002). Comprehensive human genome amplification using multiple displacement amplification. Proc Natl Acad Sci U S A.

[7] Groathouse NA, Brown SE, Knudson DL, Brennan PJ, Slayden RA (2006). Isothermal amplification and molecular typing of the obligate intracellular pathogen *Mycobacterium leprae* isolated from tissues of unknown origins. J Clin Microbiol.

[8] Kathju S, Lasken RS, Satish L, Johnson S, Stoodley P, Post JC, Ehrlich GD (2010). Multiple displacement amplification as an adjunct to PCR-based detection of *Staphylococcus aureus* in synovial fluid. BMC Res Notes.

[9] Seth-Smith HM, Harris SR, Skilton RJ, Radebe FM, Golparian D, Shipitsyna E, Duy PT, Scott P, Cutcliffe LT, O’Neill C, Parmar S, Pitt R, Baker S, Ison CA, Marsh P, Jalal H, Lewis DA, Unemo M, Clarke IN, Parkhill J, Thomson NR (2013). Whole-genome sequences of *Chlamydia trachomatis* directly from clinical samples without culture. Genome Res.

[10] McLean JS, Lombardo MJ, Ziegler MG, Novotny M, Yee-Greenbaum J, Badger JH, Tesler G, Nurk S, Lesin V, Brami D, Hall AP, Edlund A, Allen LZ, Durkin S, Reed S, Torriani F, Nealson KH, Pevzner PA, Friedman R, Venter JC, Lasken RS (2013). Genome of the pathogen *Porphyromonas gingivalis* recovered from a biofilm in a hospital sink using a high-throughput single-cell genomics platform. Genome Res.

[11] McLean JS, Lombardo MJ, Badger JH, Edlund A, Novotny M, Yee-Greenbaum J, Vyahhi N, Hall AP, Yang Y, Dupont CL, Ziegler MG, Chitsaz H, Allen AE, Yooseph S, Tesler G, Pevzner PA, Friedman RM, Nealson KH, Venter JC, Lasken RS (2013). Candidate phylum TM6 genome recovered from a hospital sink biofilm provides genomic insights into this uncultivated phylum. Proc Natl Acad Sci U S A.

